# Resident Self-Assessment and the Deficiency of Individualized Learning Plans in Our Residencies

**DOI:** 10.5811/westjem.2020.10.48615

**Published:** 2020-12-11

**Authors:** David Della-Giustina, Ali Kamran, D. Brian Wood, Katja Goldflam

**Affiliations:** * Yale School of Medicine, Department of Emergency Medicine, New Haven, Connecticut; † St. Joseph’s Medical Center, Department of Emergency Medicine, Stockton, California

## Abstract

**Introduction:**

The focus of residency training is to ensure that graduates attain a minimum level of skills and knowledge in order to be able to practice independently. While there are multiple formal methods to evaluate a resident, there is a paucity of literature that describes whether programs have residents perform individual self-assessment (ISA) with the development of individualized learning plans (ILP) to better themselves. We sought to investigate the current state of emergency medicine (EM) residency programs using ISA and determine whether these assessments are used to develop an ILP for each resident.

**Methods:**

An electronic survey was developed by educators at our institution and sent to all program leaders of United States EM residencies approved by the Accreditation Council for Graduate Medical Education. An individualized email request was sent to non-responders. Results were obtained from February–May 2019.

**Results:**

Of 240 programs we contacted, 119 (49.5%) completed the survey. Seventy-nine percent of programs reported that they had all residents perform an ISA. These were completed semiannually in 69% of the programs surveyed, annually in 19%, less than annually in 8%, and quarterly or more frequently in 4%. Of those programs requiring a resident ISA, only 21% required that all residents develop an ILP; 79% had only those residents requiring additional help or no residents develop an ILP.

**Conclusion:**

Most programs that completed the survey reported having residents complete an individual self-assessment, but there was variation in the areas assessed. The majority of programs had only lower performing, or no residents, develop an ILP based on this.

## INTRODUCTION

In 2013, the Accreditation Council for Graduate Medical Education (ACGME) released the Emergency Medicine (EM) milestones to help delineate the progress of a resident in attaining skills in each competency domain and enhancing their assessment and feedback for improvement.^1^ These milestones have become the standard for guiding resident assessment and have been used to identify individual resident improvement areas. One area that has not been standardized is a resident individual self-assessment (ISA) process. This is important, as one of the ACGME Common Program Requirements is practice-based learning and improvement, which includes constant self-evaluation and lifelong learning.^2^

Self-assessment, combined with faculty feedback, is an essential step toward improving resident performance.^3^,^4^ In EM education, other than a single oral board scenario,^3^ there is no description in the literature regarding the use of resident self-assessment paired with the development of an individualized learning plan (ILP) to promote continued resident self-improvement. In this study, we sought to investigate the current state of EM residency programs’ use of ISA and to determine further whether these assessments were used to develop an ILP for each resident.

## METHODS

A literature review was conducted by a clinical support librarian using both keywords and controlled vocabulary combining the terms for education, medical, graduate, high achiever, high performing, highly competent, rock star, intern, resident, residency, and house staff. The search was executed on January 2, 2019. The literature review encompassed seven decades from 1946 to December 31, 2018, and included the following databases: OVID Medline; OVID Embase; PubMed; the Web of Science Core Collection; Scopus; and CINAHL. A total of 1795 records with 1025 original articles were found. Based on the review of this information, we created a homegrown, 11-question survey as we were unable to find a previous survey that explored our questions. This novel survey was refined through discussion and editing by multiple EM educators at our institution to help ensure utility and comprehension. This study was approved as exempt by the institutional review board at Yale University.

The anonymous survey was sent to all ACGME-accredited EM residency leaders through the Council of Residency Directors in Emergency Medicine (CORD-EM) listserv as an anonymous link using an online survey platform (Qualtrics LLC, Provo, UT). Responses were collected from February–May 2019. An individual follow-up email was sent to the program directors who did not respond to the original request. The first question in the survey asked for the program name to ensure that no duplicate programs were included in the data analysis. At the time of the study, there were 240 ACGME-approved residency programs. Respondent characteristics and responses to survey questions are presented as counts and percentages.

## RESULTS

Of 240 programs surveyed, 119 (49.5%) completed the survey. Forty-three (36%) programs had a residency complement size of 18–30, 35 (29%) had between 31–40 residents, and 41 (34%) had more than 40 residents. Regarding resident completion of an ISA, 118 responded and 94 (79.7%) reported that they required all residents to complete an ISA, 14 (11.9%) did not require any resident to perform an ISA, and 10 (8.5%) required only those residents who needed additional help to complete an ISA. Of those programs requiring an ISA, 99 responded regarding the frequency and assessment areas for the resident ISA. The frequency of ISA completion was semiannual for 68 (69%) programs, annual for 19 (19%), less than annual for 8 (8%), and quarterly or more frequently for 4 (4%).

The percent of programs that had residents self-assess in the following categories were as follows: 90 programs required an academic ISA (90%); clinical 83 (84%); leadership 49 (50%); and other 28 (28%), with the most common free text being wellness-related in 15 (15%) programs. Academically, the 90 programs had residents self-assess in the following categories: medical knowledge 73 (81%); research 40 (44%); knowledge dissemination 32 (36%) (presentations, articles, etc); and other 13 (14%). Clinically, the 83 programs had residents self-assess in the following categories: efficiency 70 (84%); teamwork 59 (71%); management of specific medical conditions 43 (52%); presentations 35 (42%); and other 19 (22%). In leadership, the 49 programs had residents self-assess in the following categories: team leadership 35 (71%); residency leadership 33 (67%); organizational leadership 24 (49%); and other 6 (12%).

**Figure 1:**
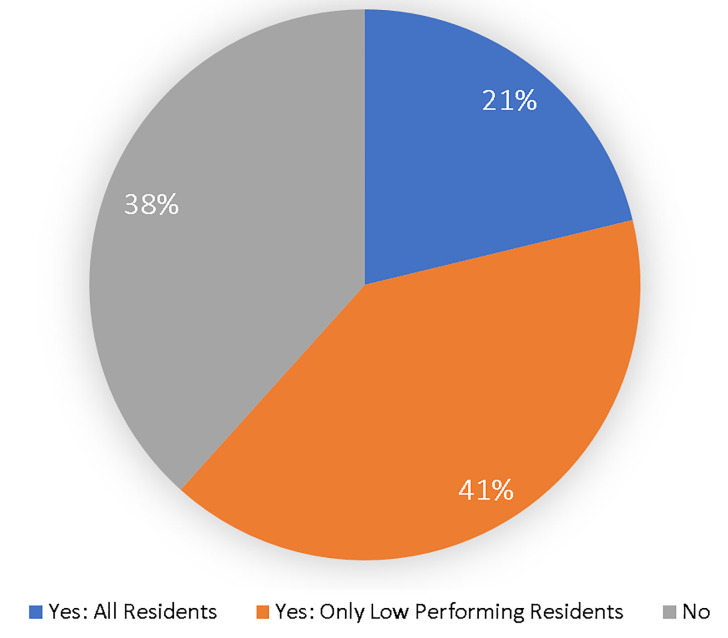
Percentage of emergency medicine residency programs requiring an individual learning plan.

Regarding the outcome of resident ISA, Figure 1 depicts how many programs required residents to develop an ILP.

## DISCUSSION

This survey is an initial appraisal regarding EM residency programs’ use of an ISA and subsequent development of an ILP. A majority of programs had residents perform an ISA on at least a semiannual basis. As would be expected, the areas of assessment focus for most programs were academic and clinical, with further subclassification into knowledge, efficiency, and team leadership. There was considerable variation in the other areas of assessment. Encouraging ISA and self-directed learning was an objective in developing the milestones in the ACGME Next Accreditation System.^5^ When performed in isolation, however, self-assessment has been found to be ineffective and inaccurate and could be considered potentially dangerous.^6^,^7^ To guide residents in developing a meaningful ISA, feedback should be used to help direct that assessment.^6.8^

In our survey, we did not inquire whether the resident ISA was used in isolation or paired with formal feedback. This is an important question, as it has been suggested that there is a poor relationship between physician self-ratings of performance and the ratings provided by external raters.^9^ Further, this inaccuracy may be worse for the least competent physicians, who overestimate their competence.^10^,^11^ A study involving EM residents demonstrated that they consistently rated themselves as better than their attendings’ assessments of them in every sub-competency assessed.^12^ This understanding of the need to pair feedback from multiple sources with an ISA should be considered in developing a standardized ISA in the future.^8^

Another finding is that only 21% of residencies have all of their residents develop an ILP. This may be interpreted that the high and even average performing residents, as defined by those programs, may be less challenged to continue their growth and development of expertise, which should be the focus of residency training. Regardless of their expertise, each resident has some area where they could further their knowledge or skills. This would be the benefit of each resident developing an ILP.

In helping residents develop an ILP, Wolf et al., suggest that this occurs with both internal and external sources of feedback regarding the resident performance under the guidance of a trusted mentor.^8^,^13^ This mentor can help guide the resident to an appropriate plan, as informed self-assessment is a flexible, dynamic process of accessing, interpreting, and responding to varied internal and external data. Alone, an informed self-assessment is characterized by multiple tensions that arise from complex interactions among competing internal and external data, multiple influencing conditions, and emotional responses to the information.^8^,^13^ The mentor can help guide the interpretation and responses to the feedback, focusing on a cogent ILP.^8^,^14^ This mentor should be engaged in the learner’s learning and improvement, aware of standards to include knowledge of curricula and level-specific standards, and skilled in facilitating and providing feedback.^14^ Guided self-assessment and self-directed learning through the development of an ILP do not mean that learners should be left on their own. Rather, they require structuring and scaffolding of learning experiences, guidance, and feedback.^14^

One consideration in the discussion on the use of ISA and the subsequent development of ILPs is the descriptive term preceding self-assessment. Sargeant uses the term “informed” self-assessment, whereas Wolff uses the term “guided” self-assessment.^8^,^13^,^14^ The term “guided self-assessment” is the more inclusive term that should be used when describing the process of a learner performing an informed self-assessment and then developing an ILP using a mentor.

## LIMITATIONS

The major limitation of this study was the response rate of just below 50%. The survey did not specify who was to take it, nor did it request the respondent’s name, so we cannot verify that a residency leader completed it. Another limitation was that we did not define several of the terms such as “lower performing resident,” “individual self-assessment,” and “formal individualized learning plan.” While this was intentional to allow each program leader to determine what they felt fit these terms, it may have confused the final results, as what one program leader considers a self-assessment may not count for another program leader. Additionally, because there is no standard definition for “lower-performing,” this lack of clarity may have led program directors to underestimate or overestimate the percentage of residents required to develop an ILP.

## CONCLUSION

Most EM programs require residents to complete some form of individual self-assessment, but there is no current standard regarding the frequency and areas assessed. Further, only a minority of programs use the ISA as a catalyst for the development of formal individualized learning plans for all of their residents. These are both areas that are open for further standardization and exploration as tools in residency education.
